# Nocturnal Heart Rate Variability in Unexplained Syncope and Sleep Apnea—The SINCOSAS Study

**DOI:** 10.3390/jcm14217864

**Published:** 2025-11-05

**Authors:** María-José Muñoz-Martínez, Manuel Casal-Guisande, Bernardo Sopeña, María Torres-Durán, Enrique García-Campo, Dolores Corbacho-Abelaira, Ana Souto-Alonso, Alberto Fernández-Villar

**Affiliations:** 1 Pulmonary Department, Hospital Universitario Álvaro Cunqueiro, 36312 Vigo, Spain; maria.luisa.torres.duran@sergas.es (M.T.-D.); alberto.fernandez.villar@sergas.es (A.F.-V.); 2 NeumoVigo I+i Research Group, Galicia Sur Health Research Institute (IIS Galicia Sur), SERGAS-UVIGO, 36312 Vigo, Spain; dcorbacho@povisa.es; 3 Faculty of Medicine and Dentistry, University of Santiago de Compostela, 15782 Santiago de Compostela, Spain; bernardo.sopena.perez-arguelles@sergas.es; 4 Department of Design in Engineering, University of Vigo, 36208 Vigo, Spain; 5 Centro de Investigación Biomédica en Red, CIBERES ISCIII, 28029 Madrid, Spain; 6 Internal Medicine Department, Hospital Clínico Universitario de Santiago de Compostela, 15782 Santiago de Compostela, Spain; 7 Cardiology Department, Hospital Universitario Álvaro Cunqueiro, 36312 Vigo, Spain; enrique.garcia.campo@sergas.es; 8 Pulmonary Department, Hospital Ribera Povisa, 36211 Vigo, Spain; 9 Pulmonary Department, Hospital Universitario de A Coruña, 15006 A Coruña, Spain; ana.souto.alonso@sergas.es; 10 School of Industrial Engineering, University of Vigo, 36310 Vigo, Spain

**Keywords:** sleep apnea, syncope, heart rate variability

## Abstract

**Background/Objectives:** Heart rate variability (HRV) reflects autonomic nervous system modulation and may be altered in both unexplained syncope and obstructive sleep apnea (OSA). However, the nocturnal autonomic patterns underlying these conditions and their coexistence remain poorly understood. This study aimed to characterize nocturnal autonomic modulation in patients with unexplained syncope, OSA, or both, compared with individuals without these conditions. **Methods:** In this multicenter, cross-sectional, comparative study, 304 adults were assigned to four groups: controls (no syncope or OSA), OSA without syncope, syncope without OSA, and syncope with OSA. Time- and frequency-domain HRV parameters were derived from overnight respiratory polygraphy and compared across groups. **Results:** OSA was associated with increased root mean square of successive differences (RMSSD) and reduced low-frequency (LF) power, indicating enhanced vagal activity and lower nocturnal sympathetic tone. Syncope was characterized by further reductions in sympathetic indices (LF and very low frequency, VLF) with increased RMSSD, suggesting blunted sympathetic reserve. Patients with both conditions exhibited a mixed autonomic profile—elevated overall HRV with concurrent reductions in both sympathetic and parasympathetic components—indicating more profound dysautonomia despite milder OSA severity. **Conclusions:** OSA and syncope show distinct nocturnal autonomic patterns, and their coexistence leads to deeper autonomic imbalance. Incorporating nocturnal HRV analysis into routine polygraphy may improve pathophysiological stratification of unexplained syncope and identify clinically significant OSA.

## 1. Introduction

Heart rate variability (HRV) refers to the fluctuations in the time intervals between consecutive heartbeats. Unlike heart rate—which measures the number of beats per minute—HRV provides insight into the modulation of the autonomic nervous system (ANS), serving as a non-invasive biomarker of its activity [1,2].

HRV can be analyzed in the time domain, frequency domain, and through non-linear methods. Preserved HRV is associated with greater physiological adaptability and better cardiovascular prognosis [3]. Conversely, reduced HRV has been linked to an increased risk of adverse events and mortality [4,5]. However, elevated HRV values do not always indicate good health: arrhythmia, such as atrial fibrillation or extrasystoles, can artificially increase HRV parameters without reflecting physiological autonomic modulation. Therefore, HRV analysis should always be interpreted in conjunction with electrocardiogram (ECG) morphology [6].

The clinical interpretation of HRV presents significant challenges, as it is influenced not only by short-term autonomic activity but also by circadian, hormonal, and long-term regulatory factors. This complexity highlights the need for standardized analysis protocols [7,8]. Although the 24 h Holter monitor remains the standard, portable devices now allow for the assessment of nocturnal HRV under more stable conditions and with reduced environmental interference [9]. During physiological sleep, parasympathetic tone predominates and sympathetic activity decreases, promoting hemodynamic rest [10]. This pattern can be altered by sleep fragmentation, as seen in obstructive sleep apnea (OSA) [11,12,13].

OSA is a prevalent condition characterized by recurrent episodes of intermittent hypoxia, micro-arousals, and sustained nocturnal sympathetic activation [14]. This imbalance has been associated with a global decrease in HRV and increased cardiovascular risk [15,16]. Nevertheless, some studies have reported mixed patterns or even increased parasympathetic activity in certain patients [13]. The apnea–hypopnea index (AHI), while widely used as a diagnostic and classificatory criterion, does not reflect autonomic load or other physiological alterations, limiting its ability to predict cardiovascular risk and other clinically relevant outcomes [17,18].

In this context, the identification of pathophysiological phenotypes—as proposed by Martínez-García et al. [19]—represents progress toward a more personalized approach to OSA. ECG recordings obtained during diagnostic polygraphy could be used to analyze HRV as a marker of autonomic modulation. Although its clinical utility is not yet fully established, HRV could help identify specific endotypes, evaluate the disorder’s impact on the ANS, and predict the response to different therapeutic strategies, including cardiovascular outcomes.

Additionally, syncope is often the result of acute autonomic dysfunction, characterized by a sudden predominance of vagal tone leading to bradycardia and hypotension [20]. In some patients with neurally mediated syncope, chronic HRV alterations have been reported, such as loss of the circadian profile and a simultaneous reduction in both sympathetic and parasympathetic activity. However, other studies have observed increased sympathetic tone in cases of unexplained syncope [21]. These findings suggest that autonomic modulation patterns may vary depending on the etiology of syncope. Furthermore, comparing studies is hampered by the lack of consistency in the metrics used to analyze HRV.

In the SINCOSAS study [22], a high prevalence of OSA was observed among patients with unexplained syncope. However, HRV parameters did not show conclusive differences, and the absence of a healthy control group limited the interpretation of the findings.

Therefore, the present study was designed with a more robust comparative approach, incorporating control groups. The objective was to characterize nocturnal HRV patterns associated with each condition and to explore whether the coexistence of OSA in patients with syncope might act as a contributing factor to autonomic dysfunction.

## 2. Materials and Methods

### 2.1. Study Design and Population

A multicenter, cross-sectional, and comparative study was conducted across three hospitals in Galicia, Spain: Hospital Universitario Álvaro Cunqueiro, Hospital Ribera Povisa de Vigo, and Complexo Hospitalario Universitario de A Coruña. Participant recruitment took place between June 2019 and May 2025. This study is part of the SINCOSAS project [22,23].

Adult patients (≥18 years) were included and classified into four groups based on the presence or absence of syncope and OSA:

- Group 1: Neither syncope nor OSA;
- Group 2: OSA without syncope;
- Group 3: Syncope without OSA;
- Group 4: Syncope and OSA.

Exclusion criteria were previously diagnosed with SA, epilepsy, or illicit drug use. Patients were recruited primarily from Cardiology (mostly those on a waiting list for subcutaneous Holter implantation), as well as from Neurology, Pulmonology, and Emergency departments. The diagnosis of syncope was made in accordance with current international clinical guidelines [20].

The study was approved by the Galician Clinical Research Ethics Committee (protocol number: 2019/048), and all participants signed an informed consent form prior to inclusion.

### 2.2. Clinical Data Collection

Demographic and anthropometric variables were collected, including age, sex, and body mass index (BMI). Relevant medical history was recorded, including arterial hypertension, ischemic or valvular heart disease, atrial fibrillation, stroke, diabetes mellitus, dyslipidemia, chronic obstructive pulmonary disease (COPD), and asthma. Smoking status (current use and pack-years) was also documented.

Additionally, symptoms suggestive of OSA were recorded, including the Epworth Sleepiness Scale, nocturnal awakenings, non-restorative sleep, and daytime fatigue. The number of reported syncopal episodes was also recorded.

### 2.3. Respiratory Polygraphy and Diagnosis of Sleep Apnea

All participants underwent home-based respiratory polygraphy using the Embletta^®^ MPR system (Natus Medical Inc., Middleton, WI, USA), with synchronized electrocardiogram recording and automatic HRV analysis. Recordings covered the period from 00:00 to 07:00 h. All signals were manually reviewed. The parameters analyzed included the apnea–hypopnea index (AHI), time with oxygen saturation below 90% (T90), desaturation index ≥ 3% (ODI3), and the number of events by type (obstructive, central, mixed apneas, and hypopneas).

OSA diagnosis was established according to the 2021 SEPAR consensus criteria [14]:

- AHI ≥ 15 events/hour, predominantly obstructive;
- AHI ≥ 5 events/hour accompanied by clinical symptoms consistent with OSA.

Severity was classified as mild (5–14.9 events/h), moderate (15–29.9/h), or severe (≥30/h).

### 2.4. Heart Rate Variability Analysis

Nocturnal HRV was assessed from continuous ECG recordings obtained during polygraphy (Embletta^®^ MPR) using 5 min segments within the 7 h nocturnal window. All respiratory parameters and the ECG recordings were manually reviewed, and artifacts were identified and excluded.

The nighttime HRV parameters analyzed were as follows:

- Time-domain: RR average (mean of RR intervals), SDNN (standard deviation of RRs), SDANN (standard deviation of RR interval averages across all segments per minute of the entire analysis interval, RMSSD (square root of the mean value of the sum of the squared differences of all successive RR intervals), SDNN index (mean of the standard deviations of all RR intervals for all segments per minute), NN50 (number of consecutive intervals varying by more than 50 ms), pNN50 (NN50 count divided by the total number of all RR intervals shown as a percentage) and the triangular index (total number of all RR intervals divided by the maximum height of the histogram).
- Frequency domain: Average total power (variance of all RR intervals), HF power (high frequency range power), VLF (very low frequency range power) and LF (low frequency range power).

Interpretation followed the recommendations of the European Society of Cardiology and the European Heart Rhythm Association [20].

Reduced HRV was defined by decreased time-domain metrics and HF (parasympathetic activity) and/or increased VLF and LF (sympathetic activity).

### 2.5. Statistical Analysis

Quantitative variables are expressed as mean ± standard deviation, and qualitative variables as frequencies and percentages. Group comparisons were performed using the Mann–Whitney U test for continuous variables and the Chi-squared test or Fisher’s exact test for categorical variables. A *p*-value < 0.05 was considered statistically significant.

Sample size was estimated based on a preliminary analysis of the first 50 patients, assuming an expected OSA prevalence of 70%, with a 95% confidence level and a 5% margin of error. To detect significant differences in HRV parameters between groups, a minimum sample of 120 patients with OSA was deemed necessary, assuming 80% statistical power and a 5% significance level, based on prior studies. Statistical analysis was performed using SPSS software version 25.0 (IBM Corp., Armonk, NY, USA).

## 3. Results

### 3.1. Characteristics of the Studied Patients

A total of 304 patients were included, with a mean age of 58.2 years and 54.6% male. The most relevant clinical characteristics and comorbidities are summarized in Table 1.

### 3.2. Comparison Between Groups

Patients were classified into four groups based on the presence or absence of syncope and OSA: Group 1 (no syncope and no OSA, *n* = 34), Group 2 (OSA without syncope, *n* = 88), Group 3 (syncope without OSA, *n* = 41), and Group 4 (syncope and OSA, *n* = 141). A comparison between these groups is presented in Table 2, Table 3 and Table 4. Table 5 presents a description of the differences observed between groups.

## 4. Discussion

This study evaluated nocturnal autonomic modulation through HRV analysis in patients with unexplained syncope, OSA, or both conditions, compared to control subjects. The aim was to identify distinctive HRV patterns that could reflect underlying autonomic dysfunction and contribute to a better pathophysiological understanding of unexplained syncope.

Our results show that both syncope and OSA are associated with HRV alterations, albeit with different profiles. While OSA patients exhibited increased time-domain parameters (RMSSD), accompanied by a reduction in sympathetic indices (LF), patients with syncope demonstrated a more pronounced reduction in nocturnal sympathetic activity.

In turn, patients with both conditions exhibited a mixed pattern, characterized by increased HRV but concurrent reductions in both sympathetic and parasympathetic components, possibly indicating functional exhaustion of the autonomic nervous system.

### 4.1. Between-Group Comparisons: Implications for Autonomic Modulation

Comparing patient groups allows identification of distinct autonomic modulation patterns in autonomic nervous system modulation during sleep. Key findings include:

- Group 1 (control) vs. Group 2 (OSA only): This comparison isolates the effect of OSA on nocturnal autonomic modulation. Although OSA patients had higher BMI and more cardiovascular risk factors, a distinct autonomic pattern was observed: significantly increased RMSSD (a vagal indicator), reduced LF component (sympathetic activity), higher nocturnal heart rate (lower mean RR), and lower total power. These findings contrast with reviews based on 24 h recordings, such as that by Sequeiros et al. [24], which report a global HRV reduction. However, studies focused specifically on nocturnal analysis—such as those by Chrysostomakis et al. [25], Bradicich et al. [26] and Salsone et al. [13]—have documented increased HRV, particularly in vagal parameters. Gula et al. [27] proposed a bimodal pattern in moderate OSA patients, with simultaneous declines in LF and HF due to combined autonomic dysfunction. Qin et al. [28] emphasized that HRV parameters may not evolve in parallel, reflecting the complex interplay between sympathetic and parasympathetic systems. Our findings support this view, suggesting that OSA is associated with altered autonomic modulation, marked by increased vagal and reduced sympathetic nocturnal activity, likely as a response to intermittent hypoxia cycles.
- Group 1 (control) vs. Group 3 (syncope without OSA): Syncope patients showed increased RMSSD (greater HRV) along with reduced LF and VLF bands, indicating lower nocturnal sympathetic activity. This pattern is consistent with Cintra et al. [29], who found decreased LF in syncope patients. Conversely, Dash et al. [21] reported elevated sympathetic activity and lower HRV in daytime recordings, which limits direct comparison. Overall, these data support the hypothesis of reduced nocturnal sympathetic reserve in patients with syncope, possibly predisposing them to vasodepressor responses during daytime.
- Group 1 (control) vs. Group 4 (syncope with OSA): Patients in Group 4 were older, with higher BMI and a greater prevalence of hypertension, dyslipidemia, diabetes, and atrial fibrillation, as well as more pronounced OSA symptoms. HRV analysis revealed increased RMSSD, SDNN, and SDNN index (higher HRV), but also reductions in LF, VLF, HF, and total power, indicating diminished sympathetic and parasympathetic activity. This mixed profile—elevated total HRV with reduced autonomic components— may reflect an altered autonomic balance with diminished modulation capacity. It is consistent with findings by Gula et al. [27] and studies by Yang et al. [11] and Mezzacappa et al. [12], which describe vagal rebound phenomena after sleep deprivation or acute stress. This autonomic alteration could reflect chronic dysautonomia triggered by nocturnal intermittent hypoxia, predisposing to vagal syncope during wakefulness.
- Group 2 (OSA) vs. Group 3 (syncope): The OSA group showed higher VLF power and lower mean RR compared to the syncope group, indicating higher nocturnal sympathetic activity. No significant differences were found in other HRV parameters. Although direct comparisons in the literature are lacking, these findings suggest that OSA is associated with predominant sympathetic activation, while syncope reflects more depressed autonomic modulation during sleep.
- Group 2 (OSA) vs. Group 4 (syncope + OSA): Despite both groups presenting with OSA, patients with syncope (Group 4) were older, had milder OSA (lower AHI), yet showed greater autonomic modulation impairment. This group exhibited elevated RMSSD (vagal indicator), while LF, VLF, and HF components were reduced, indicating deeper dysautonomia. This profile suggests that syncope contributes independently to autonomic dysfunction, beyond the severity of OSA. Salsone et al. [13] previously described significant dysautonomia even in mild or moderate OSA. In this context, increased HRV does not necessarily indicate better autonomic regulation. On the contrary, excessively high HRV may reflect dysfunctional autonomic imbalance, as previously associated with greater risk of arrhythmias and syncope [4,6,30,31]. Notably, despite having milder OSA, Group 4 patients exhibited more altered autonomic profiles than Group 2, supporting a negative synergistic effect between the two conditions.
- Group 3 (syncope) vs. Group 4 (syncope + OSA): This comparison assesses the potential additive effect of OSA on autonomic profiles in syncope patients. Although both groups shared the same clinical condition (syncope), the coexistence of OSA in Group 4 was associated with further reduction in HF band, indicating lower nocturnal parasympathetic activity. This finding supports the hypothesis that OSA contributes to deeper autonomic dysfunction in patients predisposed to syncope, even in the absence of evident differences in other clinical variables.

### 4.2. Pathophysiological and Clinical Implications

The findings of this study reinforce the concept that nocturnal autonomic modulation, assessed via HRV parameters, provides valuable insights into the functional state of the autonomic nervous system in patients with OSA, syncope, or both conditions:

- Dysautonomia and mixed pattern in OSA and syncope: The coexistence of OSA and syncope is associated with a pattern characterized by increased time-domain HRV but reduced LF (sympathetic) and HF (parasympathetic) components, suggesting an altered autonomic modulation. This complex dysautonomia may predispose individuals to vagal episodes during the day, even in the absence of active respiratory events.
- Syncope and reduced sympathetic reserve: Patients with isolated syncope showed significantly reduced nocturnal sympathetic activity. This profile aligns with the hypothesis of diminished sympathetic reserve, potentially facilitating exaggerated vasodepressor responses to stimuli such as postural changes or stress.
- OSA as a modulator of autonomic balance: Although OSA is classically associated with sympathetic activation, in this study, patients with isolated OSA exhibited increased HRV and reduced sympathetic activity, possibly reflecting a bimodal pattern with alternating bradycardia and tachycardia triggered by intermittent hypoxia. This pattern may vary according to disorder severity, sleep fragmentation, or comorbidities.
- Clinical value of nocturnal HRV analysis: HRV could serve as a complementary tool for risk stratification in patients with unexplained syncope and suspected OSA. An HRV pattern marked by reduced sympathetic and parasympathetic components may signal significant dysautonomia. Furthermore, integrating nocturnal HRV analysis into polygraphy or Holter studies may enhance diagnostic approaches in both pulmonology and cardiology.
- Integrative perspective for clinical management: These results open the possibility of using HRV as a non-invasive biomarker in the clinical approach to complex patients. They may also justify the indication for sleep studies in individuals with recurrent unexplained syncope, as well as longitudinal monitoring of autonomic modulation following CPAP treatment or comorbidity management. In fact, previous work by Chrysostomakis et al. [25] demonstrated that CPAP therapy can modulate nocturnal autonomic balance by reducing vagal tone in OSA patients, supporting the potential role of HRV in treatment monitoring.

### 4.3. Limitations and Future Directions

This multicenter, comparative study demonstrates that both unexplained syncope and OSA are associated with specific alterations in nocturnal autonomic modulation as assessed by HRV parameters. Nevertheless, several limitations must be considered.

The cross-sectional design precludes causal inference between autonomic dysfunction and the conditions studied. The analysis focused on a fixed nighttime recording (00:00–07:00 h), without sleep stage differentiation or assessment of transient events, limiting physiological interpretation. Adjustments were not made for relevant comorbidities (hypertension, atrial fibrillation, diabetes) or for medications potentially affecting HRV, introducing possible confounding. Additionally, the sample was drawn from three regional centers and analyzed using a single system (Embletta^®^ MPR), which may limit generalizability.

The predominance of male participants and the high prevalence of moderate-to-severe OSA may have influenced the autonomic patterns observed. Finally, the lack of follow-up prevents evaluation of whether OSA treatment or syncope progression modifies HRV profiles.

These limitations highlight important areas for future research: longitudinal studies assessing the prognostic value of HRV, analyses adjusted for comorbidities and pharmacologic treatments, incorporation of sleep staging, and evaluation of therapeutic impact (e.g., CPAP). Together, these efforts will help validate the clinical utility of nocturnal HRV as a biomarker in complex contexts such as unexplained syncope.

## 5. Conclusions

In this multicenter study, both unexplained syncope and OSA exhibited distinct nocturnal autonomic profiles in the analysis of HRV. While OSA was associated with increased vagal activity and reduced sympathetic tone, syncope was characterized by a more pronounced decrease in sympathetic indices. The coexistence of both conditions produced a mixed pattern with deeper dysautonomia, even in cases of mild OSA.

These findings suggest that nocturnal HRV analysis may serve as a valuable tool for the pathophysiological stratification of patients with unexplained syncope and for identifying clinically relevant OSA. Furthermore, they open the possibility of integrating HRV as a non-invasive biomarker in clinical management and in the follow-up of therapeutic interventions.

## Figures and Tables

**Table 1:** Characteristics of the study population.

Variables	Total (*n* = 304)	%/SD
Sex male	166	54.6%
Age (years)	58.16	SD 14.51
BMI (Kg/m^2^)	29.33	SD 6.05
Tobacco Never smoker Ex-smoker Active smoker	147 94 63	48.4% 30.9% 20.7%
Packs/years (in smokers and ex-smokers)	28.84	SD 26.97
Ischemic heart disease	23	7.6%
Valvular heart disease	4	1.3%
Atrial fibrillation	36	11.8%
High Blood Pressure (HBP)	113	37.2%
Stroke	5	1.6%
Diabetes	30	9.9%
Dyslipidemia	91	29.9%
Chronic obstructive pulmonary disease (COPD)	12	3.9%
Asthma	25	8.2%
Epworth Scale	9.21	SD 6.59
Daytime fatigue	154	50.7%
Nocturnal awakenings	150	49.3%
Lack of concentration	101	33.2%
Apneas observed	88	28.9%
Nocturnal choking episodes	40	13.2%
Non-restorative sleep	157	52%

**Table 2:** Comparison of clinical variables across the four study groups.

Variables	Group 1 (Without Syncope, Without SA) *n* = 34	Group 2 (Without Syncope, with SA) *n* = 88	Group 3 (with Syncope, Without SA) *n* = 41	Group 4 (with Syncope, with SA) *n* = 141	*p*-Value
Sex male	17 (50%)	45 (51.1%)	20 (48.8%)	84 (59.6%)	NS
Age (years)	50 SD (11.81)	53.57 SD (11.22)	54.20 SD (18.72)	64.14 SD (13.20)	Group 1 and 4 *p* = 0.00 * Group 2 and 4 *p* = 0.00 * Group 3 and 4 *p* = 0.00 *
BMI (Kg/m^2^)	26.36 SD (4.94)	32.49 SD (6.92)	25.79 SD (4.28)	29.11 SD (5.13)	Group 1 and 2 *p* = 0.00 * Group 1 and 4 *p* = 0.00 * Group 2 and 3 *p =* 0.00 * Group 2 and 4 *p =* 0.00 * Group 3 and 4 *p =* 0.00 *
Tobacco Never smoker Ex-smoker Active smoker	24 (70.58%) 6 (17.64%) 4 (11.88%)	40 (45.45%) 27 (30.68%) 21 (23.87%)	17 (41.46%) 11 (26.82%) 13 (31.72%)	66 (46.8%) 50 (35.46%) 25 (17.74%)	Group 1 and 2 *p =* 0.04 * Group 1 and 3 *p =* 0.03 * Group 1 and 4 *p* = 0.01 *
Packs/years (in smokers and ex-smokers)	12.8 SD (11.07)	23.63 SD (18.69)	20.54 SD (25.42)	36.95 SD (26.97)	Group 1 and 2 *p =* 0.08 Group 1 and 4 *p* = 0.04 * Group 2 and 4 *p =* 0.00 * Group 3 and 4 *p =* 0.02 *
Ischemic heart disease	2 (5.9%)	0 (0%)	3 (7.3%)	18 (12.8%)	Group 2 and 3 *p =* 0.03 * Group 2 and 4 *p =* 0.00 *
Valvular heart disease	0 (0%)	1 (1.1%)	1 (2.4%)	2 (1.4%)	NS
Atrial fibrillation	1 (2.9%)	7 (8%)	3 (7.3%)	25 (17.7%)	Group 1 and 4 *p* = 0.03 * Group 2 and 4 *p =* 0.03 *
High blood pressure (HBP)	4 (11.8%)	30 (34.1%)	9 (22%)	70 (49.6%)	Group 1 and 2 *p =* 0.01 * Group 1 and 4 *p* = 0.00 * Group 2 and 4 *p =* 0.02 * Group 3 and 4 *p =* 0.00 *
Stroke	1 (2.9%)	2 (2.3%)	0 (0%)	2 (1.4%)	NS
Diabetes	0 (0%)	5 (5.6%)	2 (4.9%)	23 (16.31%)	Group 1 and 4 *p* = 0.04 *
Dyslipidemia	5 (14.7%)	14 (15.9%)	10 (24.4%)	62 (44%)	Group 1 and 4 *p* = 0.00 * Group 2 and 4 *p =* 0.00 * Group 3 and 4 *p =* 0.02 *
Chronic obstructive pulmonary disease (COPD)	1 (2.9%)	2 (2.3%)	0 (0%)	9 (6.4%)	NS
Asthma	2 (5.9%)	6 (6.8%)	6 (14.6%)	11 (7.8%)	NS
Epworth scale	7.15 SD (6.07)	12.13 (6.77)	8.15 (6.47)	8.53 SD (6.25)	Group 1 and 2 *p =* 0.00 * Group 2 and 3 *p =* 0.00 * Group 2 and 4 *p =* 0.00 *
Daytime fatigue	10 (29.4%)	54 (61.4%)	15 (39%)	74 (52.5%)	Group 1 and 2 *p =* 0.00 * Group 1 and 4 *p* = 0.01 * Group 2 and 3 *p =* 0.01 *
Nocturnal awakenings	12 (35.3%)	46 (52.3%)	18 (43.9%)	74 (52%)	NS
Lack of concentration	7 (20.6%)	30 (34.1%)	13 (31.7%)	51 (36.2%)	NS
Apneas observed	6 (17.6%)	40 (45.5%)	9 (22%)	33 (23.4%)	Group 1 and 2 *p =* 0.00 * Group 2 and 3 *p =* 0.01 * Group 2 and 4 *p =* 0.00 *
Nocturnal choking episodes	2 (5.9%)	16 (18.2%)	3 (7.3%)	19 (13.5%)	NS
Non-restorative sleep	10 (29.4%)	56 (63.6%)	20 (48.8%)	71 (50.4%)	Group 1 and 2 *p =* 0.00 * Group 1 and 4 *p* = 0.02 * Group 2 and 4 *p =* 0.04 *
Total number of syncopes	0 SD (0)	0 SD (0)	7.63 (7.97)	8.32 SD (11.87)	Group 1 and 3 *p =* 0.00 * Group 1 and 4 *p* = 0.00 * Group 2 and 3 *p =* 0.00 * Group 2 and 4 *p =* 0.00 *

NS = ‘not significant’ = *p* ≥ 0.05; * *p* < 0.05.

**Table 3:** Comparison of respiratory variables across the four study groups.

Variables	Group 1 (Without Syncope, Without SA) *n* = 34	Group 2 (Without Syncope, with SA) *n* = 88	Group 3 (with Syncope, Without SA) *n* = 41	Group 4 (with Syncope, with SA) *n* = 141	*p*-Value
AHI	3.87 SD (2.80)	29.82 SD (22.17)	2.19 SD (1.54)	21.62 SD (16.44)	Group 1 and 2 *p =* 0.00 * Group 1 and 3 *p =* 0.03 * Group 1 and 4 *p* = 0.00 * Group 2 and 3 *p =* 0.02 * Group 2 and 4 *p =* 0.00 * Group 3 and 4 *p =* 0.00 *
ODI3	4.13 SD (3.14)	32.78 SD (22.79)	2.42 SD (2.13)	21.21 SD (16.98)	Group 1 and 2 *p =* 0.00 * Group 1 and 4 *p* = 0.00 * Group 2 and 3 *p =* 0.00 * Group 2 and 4 *p =* 0.00 * Group 3 and 4 *p =* 0.00 *
T90	0.14 SD (0.32)	13.09 SD (20.76)	1.25 SD (3.66)	14.32 SD (23.54)	Group 1 and 2 *p =* 0.00 * Group 1 and 4 *p* = 0.00 * Group 2 and 3 *p =* 0.00 * Group 3 and 4 *p =* 0.00 *
N obstructive apneas	5.06 SD (9.53)	60.31 SD (101.78)	1.85 SD (2.23)	34.16 SD (62.74)	Group 1 and 2 *p =* 0.02 * Group 1 and 3 *p =* 0.04 * Group 1 and 4 *p* = 0.00 * Group 2 and 3 *p =* 0.00 * Group 2 and 4 *p =* 0.01 * Group 3 and 4 *p =* 0.01 *
N central apneas	1.68 SD (2.61)	6.74 SD (22.07)	0.88 SD (1.88)	7.91 SD (24.40)	NS
N mixed apneas	0.24 SD (0.65)	3.51 SD (8.79)	0.2 SD (0.82)	4.62 SD (15.45)	Group 1 and 2 *p =* 0.03 * Group 2 and 3 *p =* 0.01 *
N hypopneas	16.91 SD (13.51)	118.34 SD (91.55)	10.44 SD (10.51)	90.74 SD (71.11)	Group 1 and 2 *p =* 0.00 * Group 1 and 3 *p =* 0.02 * Group 1 and 4 *p* = 0.00 * Group 2 and 3 *p =* 0.00 * Group 2 and 4 *p =* 0.01 * Group 3 and 4 *p =* 0.00 *

NS = ‘not significant’ = *p* ≥ 0.05; * *p* < 0.05.

**Table 4:** Comparison of HRV variables across the four study groups.

Variables	Group 1 (Without Syncope, Without SA) *n* = 34	Group 2 (Without Syncope, with SA) *n* = 88	Group 3 (with Syncope, Without SA) *n* = 41	Group 4 (with Syncope, with SA) *n* = 141	*p*-Value
Average RR (ms)	978.18 SD (122. 62)	919.24 SD (134.46)	1024.63 SD (174.25)	946.67 SD (152.96)	Group 1 and 2 *p =* 0.02 * Group 2 and 3 *p =* 0.00 *
SDNN (ms)	96.41 SD (34. 66)	110.34 SD (48.62)	111.30 SD (41.05)	121.55 SD (69.46)	Group 1 and 4 *p* = 0.04 *
SDNN index (ms)	69.03 SD (28.83)	86.458 SD (47.97)	81.80 SD (41.98)	99.59 SD (75.60)	NS
RMSSD (ms)	57.38 SD (39.10)	91.59 SD (90.89)	87.50 SD (64.08)	125.33 SD (134.95)	Group 1 and 2 *p =* 0.03 * Group 1 and 3 *p =* 0.01 * Group 1 and 4 *p* = 0.00 * Group 2 and 4 *p =* 0.04 *
NN50	2769.97 SD (2392.33)	3537.55 SD (3923.89)	4397.35 SD (5359.27)	4605.25 SD (5952.90)	NS
%NN50	12.84 SD (13.06)	23.38 SD (54.92)	18.96 SD (18.30)	22.97 SD (34.11)	NS
SDANN (ms)	67.80 SD (44.41)	100.54 SD (123.89)	120.43 SD (152.32)	109.19 SD (151.71)	NS
Average total power (ms^2^)	47,269.56 SD (21,839.91)	37,582.66 SD (23,582.33)	30,244.93 SD (20,945.45)	27,380.23 SD (18,453.86)	Group 1 and 2 *p =* 0.04 * Group 1 and 3 *p =* 0.00 * Group 1 and 4 *p* = 0.00 * Group 2 and 4 *p =* 0.00 *
Average VLF power (ms^2^)	23,588.05 SD (12,681.31)	19,412.22 SD (15,521.03)	13,465.10 SD (11,161.92)	12,642.97 SD (11,660.53)	Group 1 and 3 *p =* 0.00 * Group 1 and 4 *p* = 0.00 * Group 2 and 3 *p =* 0.03 * Group 2 and 4 *p =* 0.00 *
Average LF power (ms^2^)	17,858.03 SD (8596.11)	14,225.26 SD (8601.54)	11,086.03 SD (8857.22)	9645.07 SD (7753.36)	Group 1 and 2 *p =* 0.03 * Group 1 and 3 *p =* 0.00 * Group 1 and 4 *p* = 0.00 * Group 2 and 4 *p =* 0.00 *
Average HF power (ms^2^)	6170.00 SD (2391.01)	5138.67 SD (2822.85)	5173.85 SD (5602.18)	3865.66 SD (2260.14)	Group 1 and 4 *p* = 0.00 * Group 2 and 4 *p =* 0.00 * Group 3 and 4 *p =* 0.02 *
LF/HF	3.15 SD (1.9)	3.30 SD (2.97)	2.65 SD (1.93)	2.77 SD (2.40)	NS
Triangular index HRV	16.56 SD (6.27)	17.33 SD (8.37)	16.35 SD (7.99)	16.98 SD (9.39)	NS

NS = ‘not significant’ = *p* ≥ 0.05; * *p* < 0.05. HF (high-frequency power, 0.15–0.40 Hz): power in the high-frequency band reflects parasympathetic activity (vagal tone). LF (low-frequency power, 0.04–0.15 Hz): power in the low-frequency band reflects both sympathetic and parasympathetic activity. NN50: number of pairs of consecutive RR intervals that differ by more than 50 ms. pNN50: percentage of NN50 relative to the total number of RR intervals. RMSSD: root mean square of successive differences between adjacent RR intervals. RR average: mean of all RR intervals. SDANN: standard deviation of the average RR intervals calculated over 1 min segments. SDNN: standard deviation of all RR intervals. SDNN index: mean of the standard deviations of all RR intervals in 1 min segments. Total power: total variance of RR intervals, representing overall variability. Triangular index: ratio between the total number of RR intervals and the height of the histogram of their distribution. VLF (very-low-frequency power, <0.04 Hz): power in the very-low-frequency band, related to long-term regulatory mechanisms.

**Table 5:** Analysis of differences between groups.

Comparison	HRV Changes	Physiological Interpretation
Control vs. OSA	↑ RMSSD, ↓ LF, ↓ mean RR, ↓ total power	Enhanced nocturnal vagal tone with reduced sympathetic modulation
Control vs. Syncope	↑ RMSSD, ↓ LF, ↓ VLF, ↓ total power	Predominant reduction in nocturnal sympathetic activity
Control vs. Syncope + OSA	↑ RMSSD, ↑ SDNN, ↓ LF, ↓ VLF, ↓ HF, ↓ total power	Mixed autonomic pattern: high HRV but reduced sympathetic & parasympathetic indices (complex autonomic dysregulation)
OSA vs. Syncope	↑ VLF, ↓ mean RR (in OSA)	Greater nocturnal sympathetic activation in OSA compared with syncope
OSA vs. Syncope + OSA	↑ RMSSD, ↓ LF, ↓ VLF, ↓ HF, ↓ total power (Group 4)	Addition of syncope associated with deeper autonomic dysfunction, independent of OSA severity
Syncope vs. Syncope + OSA	↓ HF (in Group 4)	Further reduction in nocturnal parasympathetic activity with coexisting OSA

↑ = increased; ↓ = decreased.

## Data Availability

The data presented in this study are available upon request from the corresponding author.
